# Pelvic artery embolization versus vaginal packing for controlling vaginal bleeding in locally advanced cervical cancer: a retrospective cohort study from a tertiary cancer center in Xinjiang, China

**DOI:** 10.3389/fonc.2025.1711701

**Published:** 2026-01-06

**Authors:** Hong Yang, Fenglin Xu, Yuhan Liu, Qiuyu Chen, Yonghui Song, Yu Wu, Wei Zhong, Tingchuan Xiong, Lina You, Wukui Huang

**Affiliations:** 1 Department of Interventional Diagnosis and Treatment, The Affiliated Tumor Hospital of Xinjiang Medical University, Urumqi, Xinjiang, China; 2 Xinjiang Medical University, Urumqi, Xinjiang, China; 3 Department of Gynecologic Radiation Oncology, The Affiliated Tumor Hospital of Xinjiang Medical University, Urumqi, Xinjiang, China; 4 Department of Gynecology, The Affiliated Tumor Hospital of Xinjiang Medical University, Urumqi, Xinjiang, China; 5 Department of Traditional Chinese Medicine Oncology, The Affiliated Tumor Hospital of Xinjiang Medical University, Urumqi, Xinjiang, China

**Keywords:** cervical cancer, vaginal bleeding, pelvic artery embolization, vaginal packing, retrospective study

## Abstract

**Introduction:**

Vaginal bleeding is a frequent and potentially life-threatening complication in locally advanced cervical cancer. Pelvic artery embolization (PAE) directly occludes the bleeding arteries and allows targeted hemostasis, whereas vaginal packing (VP) relies on temporary mechanical compression. Theoretically, PAE may achieve faster and more durable bleeding control. However, few studies have compared the efficacy and safety of these two methods, limiting the early application of PAE. This study aimed to compare the effectiveness and safety of PAE versus VP, and to identify factors influencing hemostatic efficacy.

**Methods:**

This retrospective cohort study was conducted at a single tertiary cancer center in Xinjiang, China, and included patients with locally advanced cervical cancer presenting with vaginal bleeding between January 2010 and December 2024. Patients were categorized into the VP group (135 cases) and the PAE group (150 cases) based on the hemostatic intervention received. Next, we compared the hemostatic efficacy, adverse reactions, and recurrence of bleeding between the two groups. Multivariate logistic regression analysis was subsequently employed for identify the factors influencing hemostatic efficacy.

**Results:**

A total of 285 patients were included in this study. Compared with the VP group, the PAE group achieved significantly higher overall hemostatic efficacy (94.0% vs. 57.8%, P<0.001) and a lower recurrence rate of bleeding (3.3% vs. 10.4%, P=0.017). Regarding adverse reactions, the incidence of fever was higher in the PAE group (15.3% vs. 6.7%, P=0.021), whereas local infection and pelvic persistent pain were less frequent (2.7% vs. 12.6%, P=0.001; 30.7% vs. 46.7%, P=0.006). Multivariate analysis indicated that FIGO stage ≥ IIIA was independently associated with reduced hemostatic efficacy (OR=0.333, 95% CI=0.157-0.708, P=0.004), while PAE was independently associated with improved hemostatic efficacy (OR=14.026, 95% CI=6.343-31.015, P<0.001).

**Conclusion:**

PAE is more effective than VP in controlling vaginal bleeding in locally advanced cervical cancer and may be considered as an early therapeutic option when feasible. FIGO stage ≥ IIIA is identified as a risk factor for effective hemostasis, whereas PAE serves as a strong protective factor.

## 1 Introduction

Cervical cancer ranks as the fourth most prevalent malignancy in women globally, with approximately 661,021 new cases and 348,189 deaths reported (1). Globally, approximately one-third of cervical cancers are diagnosed at a locally advanced stage, with the proportion often exceeding 50% in low- and middle-income countries (2). Different guidelines provide varying definitions of locally advanced cervical cancer (LACC) according to the International Federation of Gynecology and Obstetrics (FIGO) staging system. The National Comprehensive Cancer Network (NCCN) Guidelines (3) define LACC as FIGO 2018 stages IB3 to IVA, whereas the European Society of Gynaecological Oncology (ESGO) Guidelines (4) exclude stage IIA1 from this definition. Nearly all patients at this stage present with varying degrees of vaginal bleeding and anemia, which severely affect both physical and psychological health (5). Anemia resulting from prolonged or heavy bleeding further exacerbates tumor hypoxia, reduces sensitivity to radiotherapy, and ultimately worsens patient prognosis (6). Therefore, seeking rapid and effective hemostatic methods is crucial for saving patients’ lives.

Currently, several therapeutic approaches are available for vaginal bleeding in cervical cancer, but they all have certain limitations. Internal iliac artery ligation was the standard method for controlling vaginal bleeding in the past. However, cancer recurrence or radiotherapy can cause severe deformation of the pelvic anatomy, raising concerns among physicians regarding the efficacy of this method. Furthermore, the invasiveness of the procedure increases operative difficulty and anesthetic risk in critically ill patients (7). Radiation therapy may also be employed to control vaginal bleeding. However, due to the associated risks of radiation-related complications, it is generally not considered a primary strategy for hemostasis (8, 9). In clinical practice, vaginal packing has become the most commonly used temporary hemostatic measure in most hospitals because of its simplicity and lack of requirement for specialized equipment. However, the existing literature indicates that the frequent gauze replacement often causes significant discomfort, distress, and an elevated risk of infection (6, 10). In recent years, with the advancement of interventional techniques, pelvic artery embolization has been increasingly applied for hemostasis in gynecological and obstetric conditions. It enables precise identification and occlusion of bleeding vessels, offering the advantages of minimal invasiveness and rapid recovery.

Although the hemostatic efficacy of pelvic artery embolization has been confirmed by multiple studies, most investigations of cervical cancer–related bleeding have been limited by small sample sizes (11, 12). Moreover, due to the particularity of this disease and ethical constraints, randomized controlled trials comparing pelvic artery embolization and vaginal packing are difficult to conduct. This has led to a lack of consensus on their efficacy and safety, making it difficult to choose an appropriate treatment strategy. Many medical centers over-rely on vaginal packing as the initial treatment, whereas pelvic artery embolization is only reserved as a rescue measure. Delayed intervention in some patients has led to massive hemorrhage and life-threatening consequences. Given this situation, this study retrospectively analyzed clinical data over the past 14 years to evaluate the benefits of pelvic artery embolization compared to vaginal packing in patients with locally advanced cervical cancer who present with vaginal bleeding.

## 2 Materials and methods

### 2.1 Research procedures and patients

Owing to relatively limited screening coverage and socioeconomic constraints, the burden of cervical cancer in Xinjiang remains substantial. This was a single-center retrospective study conducted at The Affiliated Tumor Hospital of Xinjiang Medical University, the largest tertiary oncology hospital in Xinjiang, China. The hospital’s Gynecologic Oncology Center has 369 inpatient beds, with 72 physicians and 99 nursing staff; the annual outpatient volume exceeds 90,000 visits, and the number of cervical cancer cases treated ranks first in Xinjiang.

**Figure 1:**
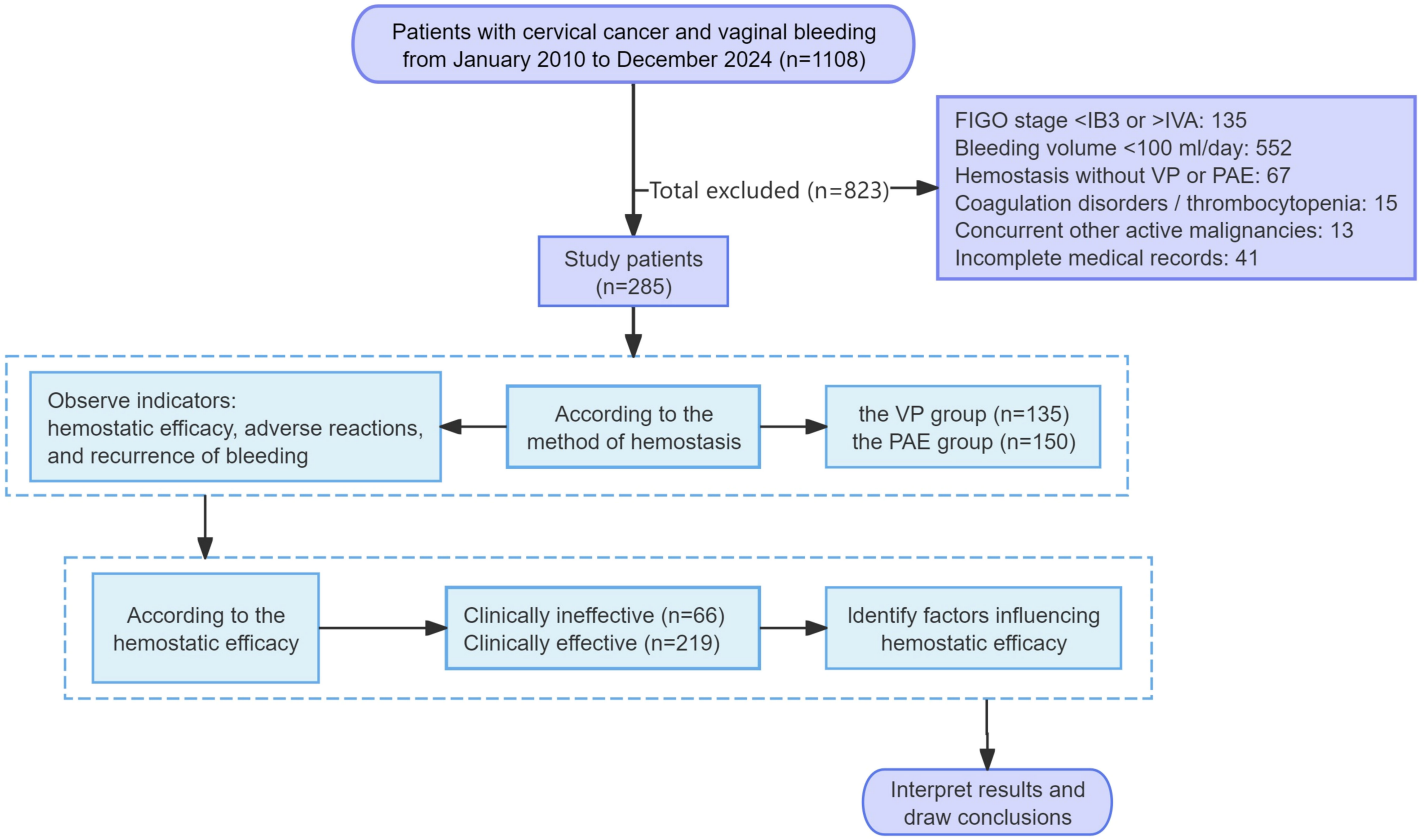
Research procedures.

We reviewed the medical records of patients with locally advanced cervical cancer with vaginal bleeding who had undergone either VP or PAE between January 2010 and December 2024. Applying consecutive sampling across the study period, we initially identified a total of 1,108 patients. After excluding 823 cases that did not meet the eligibility criteria, 285 patients were included in the final analysis. They were categorized into the PAE or VP group (PAE, *n* = 150; VP, *n* = 135) according to the hemostatic treatment they had previously received, as documented in their medical records. The choice between VP and PAE was made by the attending gynecologic oncologist according to the patient’s condition at presentation. Some patients with acute bleeding who had received temporary VP to stabilize their condition before receiving PAE were also included in the PAE group. The two groups were compared for hemostatic efficacy, adverse reactions, and recurrence of bleeding. Subsequently, all patients were evaluated for hemostatic outcomes and classified as either clinically effective or ineffective. Factors associated with hemostatic efficacy were finally identified using multivariate logistic regression analysis (**Figure 1**).

Inclusion criteria (1): pathologically confirmed locally advanced cervical cancer (FIGO stage IB3-IVA, 2018 edition) (2), bleeding volume ≥ 100 ml/day as assessed by a gynecologist (3), treatment with either VP or PAE, and (4) availability of complete clinical data. Exclusion criteria (1): received any other primary hemostatic intervention prior to or instead of VP/PAE (2), coagulation disorders or severe thrombocytopenia (3), concurrent other active malignancies (4), incomplete medical records.

### 2.2 Data sources and collection

Data were extracted primarily from the electronic medical record system. Two trained gynecologic oncology fellows independently abstracted data using a standardized form, and then discrepancies were adjudicated by a senior investigator. Variables and time points were prespecified: age, FIGO stage, tissue differentiation, tumor location, pathological type, maximum tumor diameter, HPV infection status, hematological parameters within 24 h before/after hemostasis, liver function and coagulation function within 24 h before hemostasis, blood loss volume, transfusion status, urinary tract obstruction, treatment details (VP/PAE), adverse reactions, and 3-month bleeding recurrence. Duplicate entries were removed by matching patient ID, admission date, and birth date.

“Complete clinical data” were defined as at least including available baseline demographic and tumor information, detailed treatment records, laboratory results within 24 hours before and after hemostasis, and follow-up information for at least 3 months. In this study, the incidence of missing data for the analyzed variables was very low (<2%). Given the negligible impact, patients with any missing data in the key outcome variables were excluded from the final cohort during the initial screening phase. Consequently, the analysis proceeded with a complete-case dataset, and no data imputation was applied.

This study was conducted in accordance with the Declaration of Helsinki and was approved by the Institutional Review Board of the Cancer Hospital Affiliated with Xinjiang Medical University (Approval Number: K-2024283). The requirement for informed consent was waived due to the retrospective design. To ensure confidentiality, all patient data were fully anonymized prior to analysis and stored on secure, password-protected hospital servers with access restricted to the research team.

### 2.3 Equipment and materials

The VP procedure required sterile gauze, forceps, straight scissors, a speculum, and sterile gloves. The PAE was performed using a digital subtraction angiography system (SIEMENS Artis Q). The materials included femoral artery puncture needles, vascular sheaths, catheters with matching guidewires, and contrast agents. Embolization materials included gelatin sponge particles, polyvinyl alcohol particles, and various types of coils.

### 2.4 Operating procedure

For the VP procedure, patients were placed in the lithotomy position. A sterile vaginal speculum was inserted to fully expose the cervix and upper vagina, allowing direct inspection for any active bleeding sites. Patients were instructed to relax and avoid using abdominal pressure. Under strict aseptic conditions, sterile gauze rolls were packed evenly and firmly into the vaginal vault and vagina using ring forceps to compress the bleeding surfaces. The total length of the gauze typically ranged from 1.5 to 2.0 meters, depending on vaginal capacity and the amount of bleeding. The tail of the gauze was left 2–3 cm outside the introitus to facilitate later removal, and any excess gauze was trimmed with sterile scissors. The vaginal packing was generally removed within 24 hours, and no later than 48 hours. Upon removal, bleeding status was carefully reassessed, and the patient’s response and vaginal bleeding volume were documented.

For the PAE procedure, patients were placed in the supine position with both lower limbs exposed. After routine disinfection and draping, local anesthesia was administered using subcutaneous 2% lidocaine. Right femoral artery puncture was performed using the modified Seldinger technique, followed by insertion of a 4F vascular sheath. A pigtail catheter was used for preliminary abdominal aortography to outline pelvic vascular anatomy, which was then replaced with a 4F Rosch hepatic catheter for bilateral selective internal iliac arteriography. The source of bleeding was identified based on angiographic evidence such as tumor staining or contrast extravasation. Subsequently, a microcatheter (2.0–2.8F, as appropriate) was super selectively advanced into the target branches (e.g., uterine, obturator, or internal pudendal arteries) supplying the tumor or bleeding site. The number of embolized vessels per procedure ranged from 1 to 4, depending on the extent of collateral circulation. Embolization was performed using gelatin sponge particles or polyvinyl alcohol particles (350–560 μm) until near stasis of blood flow was achieved. In cases with extensive collateral circulation, coil embolization was additionally performed to ensure complete vessel occlusion. Completion angiography was conducted to confirm successful embolization and the absence of residual bleeding. “Embolization success” was defined as complete occlusion of all angiographically visualized bleeding or tumor-feeding vessels, with no evidence of residual contrast extravasation on final angiography. After the procedure, patients were closely monitored for vital signs, pelvic pain, fever, and local ischemic symptoms. Prophylactic antibiotics and analgesics were administered as needed. Re-angiography was performed in cases of suspected recurrent bleeding during hospitalization.

### 2.5 Observation indicators

1. Bleeding volume: For significant blood loss, the weight method was adopted: bleeding volume (ml) = [wet weight of dressing after bleeding (g) - dry weight of dressing (g)]/1.05 (blood specific gravity). For minimal bleeding, the volume was roughly estimated based on the area of wet gauze. Vaginal bleeding ≤ 20 ml/day was defined as minimal (13).
2. Hemostatic efficacy: Hemostatic efficacy was categorized as follows: Significant Improvement: vaginal bleeding ceased completely within 24 hours post-treatment. Moderate Improvement: vaginal bleeding ceased within 3 days or became minimal within 7 days after treatment. No Improvement: bleeding persisted beyond 7 days with no significant improvement. The total Hemostatic Efficacy Rate = (Significant Improvement + Moderate Improvement)/total cases × 100%. Both significant improvement and moderate improvement were considered clinically effective.
3. Recurrence of bleeding: The recurrence of vaginal bleeding within 3 months after successful hemostasis was assessed via telephone follow-up or outpatient visit. Recurrence rate = Number of recurrences per group/Total patients per group.
4. Adverse reactions: The incidence of the following adverse events was recorded in both groups: local infection, pelvic persistent pain, gastrointestinal reactions, and fever. All adverse reactions were graded according to the CTCAE 5.0 criteria.

### 2.6 Statistical analysis

Categorical data were summarized as frequencies and percentages (*n*, %), with group comparisons performed using Pearson’s chi-square or Fisher’s exact test, as appropriate. Normality of continuous variables was assessed using the Shapiro–Wilk test. Continuous variables conforming to normal distribution were expressed as mean ± standard deviation and assessed via independent-samples t-tests. For non-normally distributed data, values were presented as medians with interquartile ranges M(P25–P75) and evaluated using the Mann–Whitney U test. Statistical methods were selected based on variable type and data distribution. Participants with any missing data (comprising <5% of the total cohort) were handled by listwise deletion In this study, the handling of missing data is detailed in Section 2.2.

To control for potential selection bias and confounding factors between the two treatment groups, we performed a multivariable logistic regression analysis with the primary objective of assessing the independent effect of the treatment modality itself. In this model, hemostatic efficacy was the dependent variable, and the treatment group (VP vs. PAE) was the primary independent variable of interest. The model was adjusted for baseline variables that showed a clinical relevance or imbalanced distribution. The results are presented as an adjusted odds ratio (aOR) for the treatment effect, along with its 95% confidence interval (CI). Subsequently, to comprehensively identify all independent factors associated with hemostatic efficacy, another multivariable logistic regression model was constructed. In this model, hemostatic efficacy again served as the dependent variable. All variables from the univariate analysis with a P-value < 0.2, were entered as independent variables to identify factors independently influencing hemostatic efficacy. Categorical variables (including binary, nominal, and ordinal) were appropriately coded for analysis, and continuous variables were analyzed in their measured scale.

All statistical analyses were conducted using IBM SPSS Statistics for Windows (Version 27.0. Armonk, NY: IBM Corp), and results were considered statistically significant when the two-sided P-value was under 0.05.

## 3 Results

### 3.1 Patients’ characteristics

Among the 285 patients included in this study, 150 (52.6%) were categorized into the PAE group and 135 (47.4%) into the VP group based on the hemostatic method they had received. The two groups were comparable in terms of age, FIGO stage, tissue differentiation, tumor location, pathological type, maximum tumor diameter, HPV infection status, liver function, coagulation function, blood loss volume, transfusion status, and urinary tract obstruction, with no significant differences observed (P>0.05). However, baseline hematologic parameters were generally better in the VP group compared with the PAE group (**Table 1**).

**Table 1:** Demographic and clinical characteristics of the study patients.

Variables	PAE (n=150)	VP (n=135)	P-value
Age (years)	48 (39-52)	47 (43-53)	0.289
FIGO stage			0.542
IB3	18 (12.0%)	22 (16.3%)	
IIA-IIB	29 (19.3%)	22 (16.3%)	
IIIA-IIIC	100 (66.7%)	90 (66.7%)	
IVA	3 (2.0%)	1 (0.7%)	
Tissue differentiation			
Well differentiated	28 (18.7%)	20 (14.8%)	0.664
Moderately differentiated	83 (55.3%)	80 (59.3%)	
Poorly differentiated	39 (26.0%)	35 (25.9%)	
Tumor location			0.657
outward	82 (54.7%)	69 (51.1%)	
Inward	36 (24.0%)	31 (23.0%)	
Mixed	32 (21.3%)	35 (25.9%)	
Pathological type			0.485
Squamous cell carcinoma	128 (85.3%)	119 (88.1%)	
Adenocarcinoma or Adenosquamous carcinoma	22 (14.7%)	16 (11.9%)	
Maximum tumor diameter (cm)	6.4 (5.5-7.2)	6.1 (5.0-7.1)	0.109
HPV infection status			0.703
HPV16	81 (54.0%)	77 (57.1%)	
HPV18	20 (13.3%)	20 (14.8%)	
Other types	49 (32.7%)	38 (28.1%)	
Hematological parameters			
HB (g/L)	82.5 (70.0-98.0)	94.0 (78.0-114.0)	<0.001
RBC (x10^12/L)	3.2 (2.8-3.6)	3.7 (3.0-4.1)	<0.001
HCT (%)	26.3 (23.5-31.2)	29.9 (25.1-35.1)	<0.001
PLT (x10^9/L)	266.5 (214.0-344.3)	262.0 (176.0-335.0)	0.450
Liver function			
ALT (IU/L)	11.7 (8.6-17.3)	11.5 (8.7-19.3)	0.469
AST (IU/L)	15.9 (12.5-20.1)	16.1 (13.0-20.9)	0.817
ALB (g/L)	35.0 (31.8-37.8)	36.1 (33.5-38.5)	0.071
TBil (μmol/L)	8.0 (5.5-10.9)	7.4 (5.4-9.7)	0.075
Coagulation function			
PT (s)	12.2 (11.8-12.7)	12.4 (11.8-12.8)	0.212
APTT (s)	26.0 (24.1-28.2)	26.4 (24.6-28.3)	0.321
TT (s)	17.0 (16.2-18.2)	16.8 (15.9-18.0)	0.215
FIB (g/L)	3.1 (2.7-3.9)	3.3 (2.8-3.9)	0.243
INR	1.0 (1.0-1.1)	1.1 (1.0-1.1)	0.554
Blood loss volume (ml/d)			0.362
100-200	73 (48.7%)	73 (54.1%)	
>200	77 (51.3%)	62 (45.9%)	
Transfusion status			0.383
Yes	54 (36.0%)	42 (31.1%)	
No	96 (64.0%)	93 (68.9%)	
Urinary system obstruction			0.823
Yes	35 (23.3%)	30 (22.2%)	
No	115 (76.7%)	105 (77.8%)	

HB, hemoglobin; RBC, red blood cell count; HCT, hematocrit; PLT, platelet count; ALT, alanine aminotransferase; AST, aspartate aminotransferase; ALB, albumin; TBil, total bilirubin; PT, prothrombin time; APTT, activated partial thromboplastin time; TT, thrombin time; FIB, fibrinogen; INR, international normalized ratio.

### 3.2 Treatment outcomes

**Figure 2:**
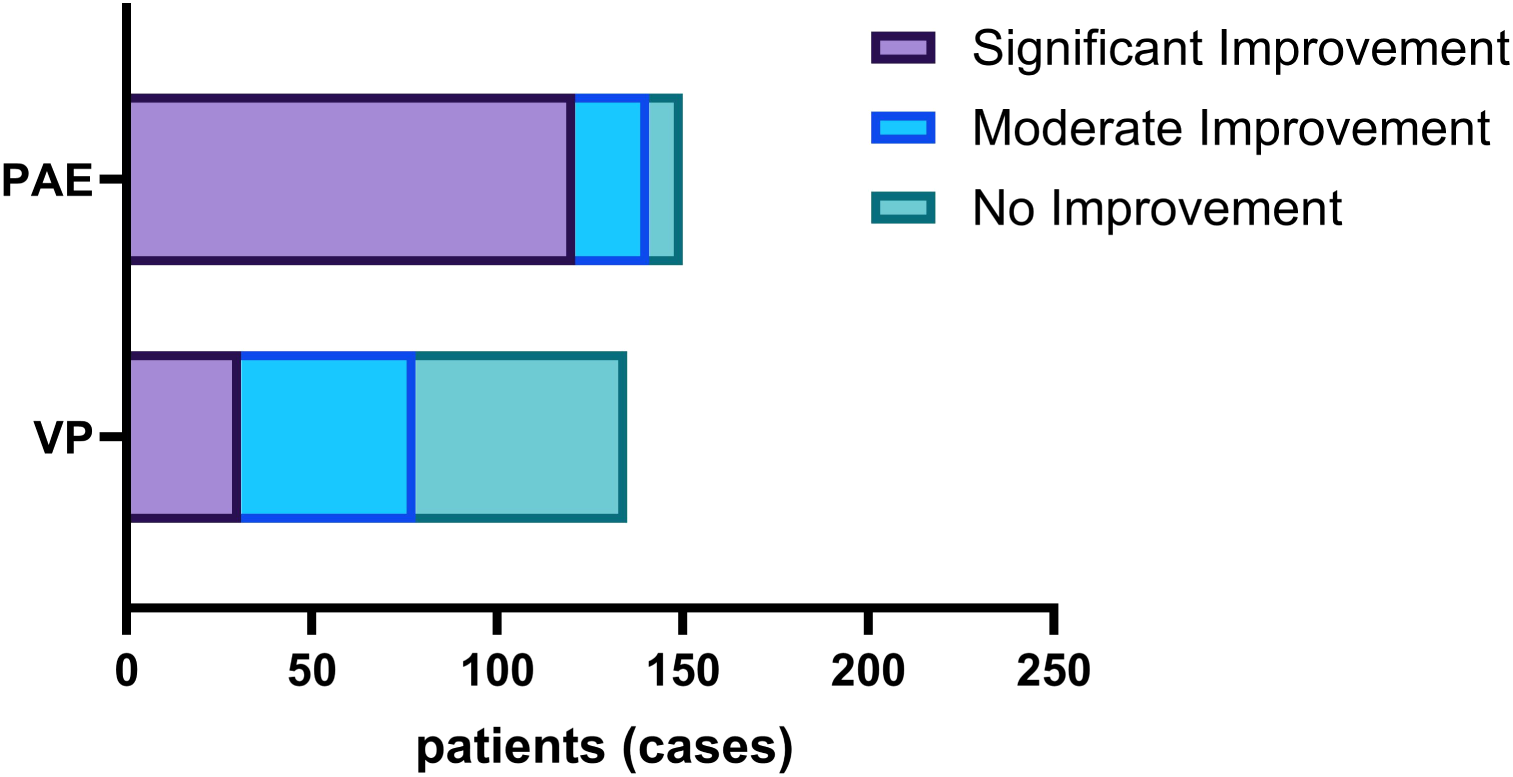
Proportion of hemostatic effectiveness.

A direct comparison of hemostatic efficacy revealed a significant difference between the two groups, with the PAE group demonstrating a markedly higher rate of total hemostatic efficacy than the VP group (94.0% vs. 57.8%, *P* < 0.001). Specifically, the proportions of “significant, moderate, and no improvement” were 80.7%, 13.3%, and 6.0%, respectively, in the PAE group, versus 23.0%, 34.8%, and 42.2% in the VP group. The PAE group accounted for a higher proportion of patients with a Significant Improvement, whereas the VP group comprised more cases in the Moderate improvement and No Improvement categories (**Figure 2**). To control for potential confounding arising from the observed imbalances in baseline HB, RBC, and HCT, a multivariable logistic regression analysis was performed. After adjustment for these confounders, PAE maintained a strong and independent association with clinically effective hemostasis compared to VP (aOR=13.084, 95%CI = 5.970 - 28.678, P < 0.001) (**Table 2**).

**Table 2:** Results of the multivariable logistic regression adjusted for confounders.

Adjusted variables	Classification	OR	95%CI	P-value
Treatment method	PAE vs. VP (Ref)	13.084	(5.970-28.678)	<0.001
HB (g/L)	–	0.981	(0.916-1.051)	0.594
RBC (x10^12/L)	–	1.721	(0.531-5.574)	0.366
HCT (%)	–	1.038	(0.763-1.412)	0.812

Post-treatment hematological parameters showed no significant change from baseline in the PAE group (P > 0.05) but decreased significantly in the VP group (P < 0.05). Regarding adverse reactions, all of their adverse reactions were below level 2 in this study (CTCAE criteria). The incidences of local infection and pelvic persistent pain were significantly higher in the VP group, whereas the incidence of fever was significantly lower (all P < 0.05). Gastrointestinal adverse events did not differ significantly between the two groups (P > 0.05). No deaths occurred during the 3 months follow-up period. Bleeding recurrence occurred less frequently in the PAE group (3.3%, 5/150) than in the VP group (10.4%, 14/135), showing a statistically significant difference (P<0.05) (**Table 3**).

**Table 3:** Treatment outcomes and adverse reactions in the two groups.

Observation indicators	PAE (n=150)	VP (n=135)	Z/χ^2^	P-value
Hemostatic Efficacy			52.388	<0.001
Significant Improvement	121 (80.7%)	31 (23.0%)		
Moderate Improvement	20 (13.3%)	47 (34.8%)		
No Improvement	9 (6.0%)	57 (42.2%)		
Total Effective Cases	141 (94.0%)	78 (57.8%)		
Hematological parameters				
HB change value (g/L)	1.0 (-5.0-9.3)	-4.0 (-10.0-2.0)*	-4.692	<0.001
RBC change value (x10^12/L)	0.0 (-0.2-0.3)	-0.2 (-0.4-0.0)*	-4.599	<0.001
HCT change value (%)	0.3 (-1.7-2.3)	-1.4 (-3.5-0.6)*	-4.460	<0.001
PLT change value (x10^9/L)	-0.5 (-25.5-26.0)	-5.0 (-40.0-24.0)	-1.309	0.191
Adverse Reactions				
Local infection	4 (2.7%)	17 (12.6%)	10.256	0.001
Pelvic persistent pain	46 (30.7%)	63 (46.7%)	7.701	0.006
Gastrointestinal reactions	10 (6.7%)	8 (5.9%)	0.066	0.797
Fever	23 (15.3%)	9 (6.7%)	5.354	0.021
Recurrence of bleeding	5 (3.3%)	14 (10.4%)	5.655	0.017

*indicated statistically significant differences compared with pre-treatment levels, P<0.05. Significant improvement = bleeding cessation ≤ 24 h after treatment; Moderate improvement = bleeding cessation ≤ 3 days or minimal bleeding ≤ 7 days; No improvement = bleeding persisting > 7 days. Total hemostatic efficacy rate = (Significant improvement + Moderate improvement) /total cases × 100%; “Pelvic persistent pain” was defined as persistent pain lasting more than 3 days after treatment.

### 3.3 Influencing factors of hemostatic efficacy

All patients were categorized into clinically effective (n=219) and clinically ineffective (n=66) groups based on hemostatic outcome. **Table 4** presents both univariate and multivariate logistic regression analyses of factors associated with hemostatic effectiveness. To avoid omitting potentially important predictors, variables with a P-value<0.2 in univariate analysis were subsequently incorporated into the multivariate logistic regression model. The results indicated that FIGO stage and treatment method were independent factors influencing hemostatic efficacy. FIGO stage ≥ IIIA was associated with lower hemostatic efficacy (OR = 0.333, 95% CI = 0.157-0.708, P = 0.004). Conversely, the use of PAE was associated with higher hemostatic efficacy (OR = 14.026, 95% CI = 6.343-31.015, P<0.001).

**Table 4:** Univariate and multivariate analysis of factors influencing hemostatic effectiveness.

Variables	Univariate OR (95% CI)	P-value	Multivariate OR (95% CI)	P-value
Age (years)				
<48	Reference			
≥48	1.335 (0.768-2.323)	0.306		
FIGO stage				
≤IIB	Reference		Reference	
≥IIIA	0.443 (0.228-0.864)	0.017	0.333 (0.157-0.708)	0.004
Tissue differentiation				
Well differentiated	Reference			
Moderately differentiated	1.128 (0.521-2.441)	0.760		
Poorly differentiated	0.750 (0.323-1.741)	0.504		
Tumor location				
outward	Reference			
Inward	0.679 (0.351-1.313)	0.250		
Mixed	0.932 (0.465-1.867)	0.843		
Pathological type				
Squamous cell carcinoma	Reference			
Adenocarcinoma or Adenosquamous carcinoma	1.151 (0.500-2.648)	0.741		
Maximum tumor diameter (cm)	0.997 (0.850-1.169)	0.969		
HPV infection status				
HPV16	Reference			
HPV18	1.053 (0.460-2.412)	0.902		
Other types	1.024 (0.551-1.905)	0.939		
Hematological parameters				
HB (g/L)	0.994 (0.981-1.007)	0.340		
RBC (x10^12/L)	0.934 (0.629-1.386)	0.733		
HCT (%)	0.981 (0.938-1.026)	0.398		
PLT (x10^9/L)	0.998 (0.996-1.001)	0.149	0.999 (0.996-1.001)	0.298
Liver function				
ALT (IU/L)	0.986 (0.969-1.002)	0.093	0.989 (0.969-1.008)	0.256
AST (IU/L)	0.995 (0.974-1.015)	0.604		
ALB (g/L)	0.964 (0.908-1.024)	0.231		
TBil (μmol/L)	1.033 (0.963-1.108)	0.365		
Coagulation function				
PT (s)	1.123 (0.840-1.500)	0.434		
APTT (s)	1.004 (0.930-1.083)	0.924		
TT (s)	1.053 (0.915-1.211)	0.472		
FIB (g/L)	1.000 (0.744-1.342)	0.998		
Blood loss volume (ml/d)				
100-200	Reference		Reference	
>200	0.631 (0.362-1.099)	0.104	0.551 (0.283-1.073)	0.079
Transfusion status				
No	Reference		Reference	
Yes	0.664 (0.377-1.172)	0.158	0.570 (0.281-1.157)	0.119
Urinary system obstruction				
No	Reference			
Yes	0.901 (0.472-1.719)	0.751		
Treatment method				
VP	Reference		Reference	
PAE	11.449 (5.379-24.370)	<0.001	14.026 (6.343-31.015)	<0.001

### 3.4 *Post hoc* power analysis

Because this was a retrospective study including all consecutive eligible patients, no *a priori* sample-size calculation was performed. To assess whether the sample size was sufficient to detect the observed difference in hemostatic efficacy between the PAE and VP groups, a *post hoc* power analysis was conducted using G*Power version 3.1.9.2 (Z test for two independent proportions, two-sided α = 0.05; n_1_ = 150, n_2_ = 135; p_1_ = 0.94, p_2_ = 0.578). The achieved statistical power was >99.9%, indicating that the study sample size was adequate to detect the observed effect.

## 4 Discussion

Vaginal bleeding is a common and sometimes life-threatening complication in patients with locally advanced cervical cancer, and effective hemostatic management is critical for stabilizing their clinical condition and enabling subsequent oncologic treatment. This retrospective study demonstrated that PAE achieved a markedly higher hemostatic efficacy and a lower recurrence rate compared with conventional VP, particularly in early hemostasis (within 24 hours). These findings reinforce the clinical value of interventional radiology techniques as a rapid and reliable option for bleeding control in this population.

PAE achieves hemostasis by selectively occluding tumor-feeding arteries under angiographic guidance, thereby directly eliminating the arterial blood supply to the bleeding site and achieving rapid bleeding control (12). This mechanism allows PAE to target deep or multiple bleeding vessels that cannot be effectively compressed by conventional methods. In contrast, VP relies solely on mechanical compression to reduce local blood flow and promote coagulation, which is less effective for bleeding originating from deeper or collateral vessels (14). These mechanistic differences explain the markedly higher hemostatic efficacy and faster bleeding control observed with PAE compared to VP.

It is noteworthy that the hemostatic efficacy rate in the PAE group in this study was consistent with previous single-arm studies, but the recurrence rate was lower (15). Several factors may account for this discrepancy. First, this study used selective artery embolization, whereas most earlier studies employed unilateral or bilateral uterine artery embolization. Although the uterine arteries are the primary blood supply to cervical cancer, tumors at advanced stages often develop extensive collateral circulation. Other branches of the internal iliac artery, including the obturator and internal pudendal arteries, may also supply blood to the tumor (16). Therefore, embolizing only the uterine arteries may miss other bleeding branches, increasing the risk of recurrence. Second, 59.3% (89/150) of patients in this study received concurrent arterial infusion chemotherapy during embolization, which may be another reason for the lower recurrence rate in the PAE group. In our clinical practice, this combined approach is used selectively to enhance local control, typically in patients with a higher tumor burden and good treatment tolerance. The chemotherapeutic dose administered intra-arterially was approximately one-third of that used for systemic intravenous chemotherapy, thus primarily serving an adjunctive rather than a dominant hemostatic role. The principal mechanism of bleeding control remained mechanical occlusion of the feeding vessels by embolization. Based on this consideration, at the outset of the study design, whether or not combined with intra-arterial chemotherapy, embolization was regarded as the core of hemostasis. Therefore, the two types of patients were classified into the PAE group. This inevitably introduced confounding bias. However, because the number of bleeding-recurrence events in the PAE group was relatively small (5 events in total, accounting for 3.3%), it is difficult to reliably determine whether the lower recurrence rate observed in the PAE cohort was truly attributable to intra-arterial chemotherapy or merely represents a chance finding, which is a limitation of this study. We speculate that, because chemotherapy drugs require a certain period of time to exert their therapeutic effects, the addition of local chemotherapy is unlikely to have substantially altered the short-term hemostatic outcome when comparing PAE with VP. However, platinum-based chemotherapy can significantly reduce tumor size and burden, thereby indirectly decreasing vaginal bleeding in the longer term. Accordingly, part of the observed difference in recurrence rates between PAE and VP may be attributable to this imbalance rather than to embolization alone. Overall, whether intra-arterial chemotherapy should be used during embolization for hemostasis, and its specific impact on hemostatic efficacy and recurrence rates, remains undetermined and warrants further investigation with a larger sample size.

Postembolization syndrome is a common complication after PAE and includes fever, pain, nausea, and leukocytosis. These symptoms are mainly caused by local ischemia following embolization and the absorption of necrotic material. With close observation and symptomatic treatment, they gradually resolve within a few days (17, 18). In this study, 55.4% of patients in the PAE group experienced the situations mentioned above. Pelvic persistent pain accounted for the highest proportion (30.7%), which is consistent with previous studies (19, 20). In the VP group, pelvic persistent pain was also the most frequent adverse event (46.7%), with a higher incidence than in the PAE group (P = 0.006). PAE-related pain is typically ischemic in nature and tends to decrease as the patient adapts and tissue reperfusion occurs. In contrast, patients undergoing VP often experience repeated discomfort and persistent irritation due to regular gauze replacement every 12–48 hours, which can significantly reduce their quality of life. The VP group also showed a higher local infection rate than the PAE group (12.6% vs 2.7%; P = 0.001), indicating that gauze retention and repeated replacement may increase the risk of infection. Although fever occurred more often in the PAE group than in the VP group (15.3% vs. 6.7%; P = 0.021), symptoms resolved in both groups after supportive treatment. In addition, angiography-related complications such as puncture-site hematoma, pseudoaneurysm, and arteriovenous fistula, as well as rare events including uterine rupture and ischemic necrosis of the bladder or rectum, may occur during PAE. These complications are typically associated with inadvertent embolization of non-target vessels (21, 22). None of these situations was observed in this study. With a thorough understanding of vascular anatomy and proficient embolization techniques, the risk of such complications can be minimized. The results above indicated that both PAE and VP are relatively safe treatments for vaginal bleeding, with no serious complications observed. However, compared with VP, PAE can reduce persistent pain and the incidence of local infection to a certain extent, thereby improving patients’ quality of life.

This study also found that, after adjustment for confounding factors, patients with FIGO stage ≥IIIA had significantly lower odds of achieving effective hemostasis compared with those with stage ≤IIB. The likelihood of effective hemostasis in stage ≥IIIA patients was approximately one-third that of patients with stage ≤IIB (OR = 0.333, 95%CI=0.157–0.708, P = 0.004). This may be explained by the vascular complexity of advanced tumors. Malignant tumors that invade adjacent normal tissues primarily rely on neovascularization. These newly formed blood vessels are fragile and prone to rupture, leading to massive hemorrhage. At later tumor stages, erosion and destruction of surrounding blood vessels are more extensive, accompanied by the formation of abnormal neovascularization and abundant collateral circulation. These factors collectively make complete and sustained hemostasis more difficult to achieve in advanced stage patients (23). To date, no studies have specifically analyzed the factors influencing the hemostatic efficacy in vaginal bleeding associated with cervical cancer, and the precise mechanisms require further exploration.

Although this study provides evidence for the management of vaginal bleeding, some limitations remain. Firstly, this study adopted a retrospective design, which inevitably introduces selection bias, as treatment choice was not randomized but based on clinical judgment. Although multivariable logistic regression was applied to control for confounding factors, unmeasured or residual bias may still exist. Secondly, the follow-up period was relatively short, and the long-term hemostatic efficacy of PAE requires further validation. Thirdly, diagnostic and treatment protocols may vary slightly across medical centers. As this study was limited to patients at a single institution, the generalizability of the findings may be restricted. Future large-scale, multicenter studies are warranted to validate these results.

## 5 Conclusion

Compared with conventional vaginal packing, pelvic artery embolization was associated with superior hemostatic efficacy and a lower recurrence rate for controlling vaginal bleeding in locally advanced cervical cancer, while maintaining a favorable safety profile. Additionally, clinicians should also exercise heightened vigilance in patients with FIGO stage IIIA or higher, as these individuals are at greater risk of hemostatic failure. These findings suggest that PAE may represent a more effective therapeutic approach, particularly for these high-risk patients.

## Data Availability

The original contributions presented in the study are included in the article/**Supplementary Material**. Further inquiries can be directed to the corresponding author.
